# Pattern of care of prostate cancer patients across the Martinique: results of a population-based study in the Caribbean

**DOI:** 10.1186/s12885-018-5047-5

**Published:** 2018-11-16

**Authors:** Clarisse Joachim, Jacqueline Veronique-Baudin, Stephen Ulric-Gervaise, Jonathan Macni, Thierry Almont, Olivier Pierre-Louis, Lidvine Godaert, Moustapha Drame, Jean-Luc Novella, Karim Farid, Vincent Vinh-Hung, Patrick Escarmant

**Affiliations:** 1 CHU Martinique, UF1441 Registre des cancers de la Martinique, Pôle de Cancérologie Hématologie Urologie Pathologie, CS 90632, 97200 Fort-de-France, Martinique France; 2 CHU Toulouse Paule de Viguier, Groupe de recherche en fertilité humaine EA 3694, Toulouse, France; 3 Groupe d’Étude, de Formation et de Recherche en Andrologie, Urologie et Sexologie Médecine de la Reproduction, Toulouse, France; 4 CHU Martinique, Pôle de Cancérologie, Hématologie Urologie Pathologie, 97200 Fort-de-France, Martinique France; 5 CHU de Martinique, Pôle de Gériatrie, 97200 Fort-de-France, Martinique France; 6 CHU de Reims, Pôle Recherche et Santé publique, 51100 Reims, France; 7 CHU de Reims, Pôle de Gériatrie, 51100 Reims, France; 8 CHU Martinique, Pole d’imagerie Médicale Service de Médecine nucléaire, 97200 Fort-de-France, Martinique France; 9 CHU MARTINIQUE, Pôle de Cancérologie Hématologie Urologie Pathologie, 97200 Fort-de-France, Martinique France

**Keywords:** Epidemiology, Caribbean, Diagnosis, Prostate cancer, Cancer registry

## Abstract

**Background:**

The French West-Indies rank first for both prostate cancer incidence and mortality rates. Analyzing diagnostic and therapeutic procedures among patients with prostate cancer, using data from a population-based cancer registry, is essential for cancer surveillance and research strategies.

**Methods:**

This retrospective observational cohort study was based on data from the Martinique Cancer Registry. Records of 452 patients diagnosed with prostate cancer in 2013 were retrieved from the registry. Data extracted were: socio-demographic and clinical characteristics, circumstances of diagnosis, PSA level at diagnosis, Gleason score and risk of disease progression. Stage at diagnosis and patterns of care among prostate cancer patients were analyzed.

**Results:**

Mean age at diagnosis was 67 ± 8 years; 103 (28.5%) were symptomatic at diagnosis. Digital rectal exam was performed in 406 (93.8%). Clinical stage was available in 385 (85.2%); tumours were localized in 322/385 (83.6%). Overall, 17.9% were at low risk, 36.4% at intermediate and 31.9% at high risk; 13.8% were regional/metastatic cancers. Median PSA level at diagnosis was 8.16 ng/mL (range 1.4–5000 ng/mL). A total of 373 patients (82.5%) received at least one treatment, while 79 (17.5%) had active surveillance or watchful waiting. Among patients treated with more than one therapeutic strategy, the most frequent combination was external radiotherapy with androgen deprivation (*n* = 102, 22.6%).

**Conclusions:**

This study provides detailed data regarding the quality of diagnosis and management of patients with prostate cancer in Martinique. Providing data on prostate cancer is essential for the development of high-priority public health measures for the Caribbean.

## Background

In 2012, prostate cancer (PCa) was the second most common cancer in men worldwide, with wide variation in incidence rates, which can be explained by differences in detection practices, treatment availability, lifestyle, environmental and genetic factors [1]. PCa incidence and mortality rates are high in the Caribbean, with an estimated 79.8 and 29.0 cases per 100,000 men respectively in this predominantly black population, and PCa is the leading cause of cancer deaths [2–5].

In the Caribbean, the French West-Indies rank first for both PCa incidence and mortality rates, which have been suggested to be partially related to the high prevalence of some gene polymorphisms and environmental endocrine disruptors [6–8]. Higher tumor grade is also reported in African-Caribbean populations and a higher risk of metastatic progression among black men; studies on PCa management are a huge challenge in our region to investigate and explain the observed higher incidence [9–13].

The Martinique Cancer Registry (MCR) is a population based cancer registry that collects population data in Martinique, an overseas region of France with a population of 386,486 inhabitants. The MCR is one of the only two French population-based cancer registries (PBCR) among the 30 nations and territories of the Caribbean [14].

Between 2010 and 2014, the MCR reported 535 new cases of PCa per year with an incidence rate of 161 per 100,000 men, placing Martinique among the regions with the highest PCa rates in the world [15, 16]. For men diagnosed during this period, 5-year survival was 97.5% with a world-standardized mortality rate of 23.5 per 100,000 [17].

In Martinique, there is no screening program for PCa, treatments can vary according to health care providers, patients, organizational resources and facilities. The manner in which these factors interact greatly shape the patient’s care plan.

The burden of PCa will increase in the future, due to the known association between older age and cancer. The management of elderly prostate cancer patients requires the assessment of overall health status and life expectancy based on the combination of age and comorbidity [18], to take into account the risk of dying from prostate cancer or another cause, the risks of treatment, and patient preferences [18–20].

Although national and international guidelines for PCa management exist, large-scale surveys are needed in the Caribbean, in view of the high incidence of this cancer and the predisposition of its populations to PCa [21–24]. In this regard, accurate data from PBCRs is important to evaluate the burden of disease, and, when complemented with detailed assessment of care and management, can provide important information on the health system organisation. To date, few studies have investigated the clinical stage at diagnosis and the management patterns of PCa in the Caribbean zone based on data from population-based cancer registries [1, 25–29]. The aim of this study was therefore to analyze diagnostic and therapeutic procedures among patients with PCa and the clinical characteristics according to age, in Martinique in 2013, using data from the MCR.

## Methods

### Study population

Population-based cancer registries perform continuous and exhaustive recording of all new cancer cases occurring in the population resident in a given area, regardless of where the diagnosis or the treatment takes place. The registries meet a twofold objective, namely description and surveillance of cancer risk, and secondly, research based on the analysis of the data thus collected, or by means of cross-sectional surveys [30, 31]. Observation and follow-up of cancer cohorts are a complement to surveillance of cancer incidence, and make it possible to describe incidence and prognosis in greater detail. This was a population-based study based on data from the MCR. We included data from all patients who were diagnosed with PCa (ICD10: C61) in 2013 in private and public hospitals. Patients with a history of, or recurrence of PCa, or those treated for another cancer were excluded from the study.

### Data collection

Data were recorded in the PBCR database of Martinique in strict conformity with the international standards laid down by the International Agency for Research on Cancer, the French FRANCIM network, and the European Network of Cancer Registries. Quality control of all recorded cases in the MCR was performed in accordance with international guidelines for cancer registries. The following variables were recorded and retrieved for all cases: date of diagnosis, patient’s age, symptoms at diagnosis and complementary imaging exams performed.

Total blood prostate-specific antigen (PSA) levels at the time of diagnosis were grouped into five categories: 1) <  4 ng/mL, 2) 4.01–10 ng/mL, 3) 10.01–20 ng/mL, 4) 20.01–100 ng/mL, 5) > 100 ng/Ml. Clinical stage at diagnosis was classified into localized (T1N0M0 - T2N0M0 and T3aN0M0) versus locally advanced (T3b/T4N0M0) and regional/metastatic group (N+/M+) based on the TNM classification, 7th edition (2010). The modified Gleason score proposed by the International Society of Urological Pathology [32] was used to define five prognostic groups, namely Grade Group 1 (formerly Gleason score ≤ 6); Grade Group 2 (Gleason score 3 + 4 = 7); Grade Group 3 (Gleason score 4 + 3 = 7); Grade Group 4 (Gleason score 4 + 4 = 8; 3 + 5 = 8; 5 + 3 = 8); and Grade Group 5 (Gleason scores 9–10). Patients were stratified into three localized risk groups according to the French Guidelines [21], and grouping the regional and metastatic categories because of the low number of patients, instead of eight groups according to the NCCN Guidelines [33]:

- Low risk: (1) T1a, T1b, T1c, or T2a and N0, M0; and (2) PSA level < 10 ng/mL; and (3) Gleason score 6 or less;
- Intermediate risk: (1) T2b and N0, M0; or (2) PSA level between 10 and 20 ng/mL; or (3) Gleason score 7;
- High risk: (1) cancer stage T2c, T3a, T3b or T4, N0, M0; or (2) PSA level greater than 20 ng/mL; or (3) Gleason score between 8 and 10;
- Regional/ Metastatic cancer: any N1 M0, or M1.

Radiological diagnostic tests and treatment within 24 months of diagnosis were included in the analysis. Radiotherapy, brachytherapy, prostatectomy, androgen deprivation therapy, active surveillance/watchful waiting (no active treatment…) received by each patient were retrieved from the database and medical records.

### Statistical analysis

Patient characteristics are described as mean ± standard deviation for quantitative variables, and number (percentage) for qualitative variables. Comparisons were performed using the Student t and Chi square or Fisher’s exact tests, as appropriate. Missing data were analysed for each variable.

We analyzed socio-demographic characteristics (age at diagnosis and area of residence at the time of diagnosis) and clinical factors such as symptoms at diagnosis, diagnostic procedures, clinical stage at diagnosis and therapeutic management. Patients were classified into three age groups (< 65 years, 65–74 years and ≥ 75 years) to enable analysis of variations in clinical characteristics according to age. All analyses were performed using SAS version 9.2. (SAS Institute Inc., Cary, NC, USA). For all analyses, a *p* value < 0.05 was considered statistically significant.

## Results

### Demographic and diagnostic characteristics

In total, 473 new cases of PCa were diagnosed in Martinique in 2013. We excluded 21 patients because they were multiple tumour cases. Finally, 452 patients were included in the final analysis of this paper. Table 1 presents the baseline characteristics of the study population. Almost 60% (*n* = 269) were aged 65 years and older (median 67, range 45 to 86 years).

**Table 1:** Demographic and clinical characteristics among patients with prostate cancer (*N* = 452) according to age group, from the Martinique Cancer Registry, 2013

Characteristics	All		≤ 64 years		65–74 years		≥ 75 years		*P*
	n	%	n	%	n	%	n	%	
	452	100.0	183	40.5	178	39.4	91	20.1	
Symptoms at diagnosis									0.024
Yes	103	28.5	35	23.8	38	26.8	30	41.1	
No	259	71.5	112	76.2	104	73.2	43	58.9	
Unknown	90	–	36	–	36	–	18	–	
Clinical stage at diagnosis									0.0837
T1N0M0	168	43.6	78	49.1	63	41.4	27	36.5	
T2N0M0	121	31.4	46	28.9	48	31.6	27	36.5	
T3/T4N0M0	43	11.2	22	13.8	15	9.9	6	8.1	
N+/M+	53	13.8	13	8.2	26	17.1	14	18.9	
Unknown	67	–	24	–	26	–	17	–	
Prostate Specific Antigen (ng/ml)									0.17
< 4	19	4.2	12	6.6	4	2.2	3	3.3	
4 ≥ PSA > 10	263	58.2	115	62.8	102	57.3	46	50.5	
10 ≥ PSA > 20	95	21.0	30	16.4	43	24.2	22	24.2	
20 ≥ PSA > 100	46	10.2	17	9.3	17	9.6	12	13.2	
≥ 100 ng/ml	29	6.4	9	4.9	12	6.7	8	8.8	
Gleason grade group									0.003
Group 1 (3 + 3)	152	34.0	79	43.7	56	31.8	17	18.9	
Group 2 (3 + 4)	117	26.2	46	25.4	45	25.5	26	28.9	
Group 3 (4 + 3)	84	18.8	31	17.1	36	20.5	17	18.9	
Group 4 (8)	48	10.7	13	7.2	19	10.8	16	17.8	
Group 5 (9 or 10)	46	10.3	12	6.6	20	11.4	14	15.5	
Unknown	5	–	2	–	2	–	1	–	
Risk groups									0.0129
Low	69	17.9	40	25.2	22	14.5	7	9.5	
Intermediate	140	36.4	57	35.8	58	38.1	25	33.8	
High	123	31.9	49	30.8	46	30.3	28	37.8	
Regional/metastatic	53	13.8	13	8.2	26	17.1	14	18.9	
Unknown	67	–	24	–	26	–	17	–	

In total, 103 patients (28.5%) presented clinical symptoms at diagnosis, mainly urinary or genital (87.4% of those with symptoms).

Digital rectal exam (DRE) was performed in 406 patients (93.8%) by urologists or family physicians, and led to suspicion of cancer in 54.6% of cases.

Patients in the oldest age group (75+) more often presented with symptoms as compared to younger patients. Analysis of symptoms according to age showed a statistically significant difference in the rate of symptoms across age groups (*p* = 0.024).

Clinical stage at diagnosis was available for analysis in 385 patients (85.2%). The tumors were localized in 322 (83.6%), and locally advanced in 10 patients (2.6%). A total of 53 (13.8%) had node positive and distant metastases at diagnosis. We found that 79.2% of patients with localized disease were at intermediate or high risk (263 / 332).

There was an increasing proportion of advanced tumours with increasing age, although this association was not statistically significant. We observed a statistically significant increase in Gleason score and in the risk of disease progression for the oldest patients (≥ 65 years). For example, among those aged 75 years and older, 37.8% were in the highest risk group, as compared to only 30.8% among those younger than 65 years (*p* = 0.0129). Finally, we observed that PSA level at diagnosis was significantly higher in patients with advanced cancer (*p* < 0.0001) (Table 1).

**Fig. 1:**
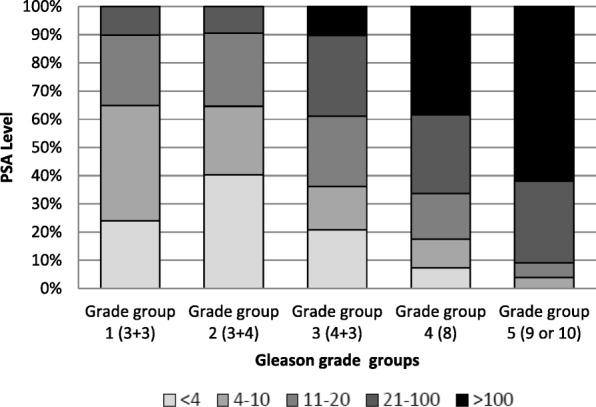
Analysis of stage of PSA group by Gleason grade group

Similar findings were observed for Gleason score, i.e., increasing PSA levels at diagnosis were associated with increasing Gleason score (Fig. 1, Table 2).

**Table 2:** Gleason grade group according to PSA level at diagnosis (ng/ml) among patients with prostate cancer, (*N* = 452), from the Martinique Cancer Registry, 2013

	< 4 (*n* = 19)		4 ≥ PSA > 10 (*n* = 263)		10 ≥ PSA > 20 (*n* = 95)		20 ≥ PSA > 100 (*n* = 46)		≥ 100		*P*
									(*n* = 29)		
	n	%	n	%	n	%	n	%	n	%	
Gleason grade group (*N* = 447)											–
Group 1 (3 + 3)	5	26.3	116	44.8	26	27.4	5	11.1	–	–	
Group 2 (3 + 4)	9	47.4	74	28.6	29	30.5	5	11.1	–	–	
Group 3 (4 + 3)	4	21.0	40	15.4	24	25.2	13	28.9	3	10.3	
Group 4 (8)	1	5.3	19	7.3	11	11.6	9	20.0	8	27.6	
Group 5 (9 or 10)	–	–	10	3.9	5	5.3	13	28.9	18	62.1	
Unknown	–	–	4	–	–	–	1	–	–	–	

### Treatment characteristics

**Fig. 2:**
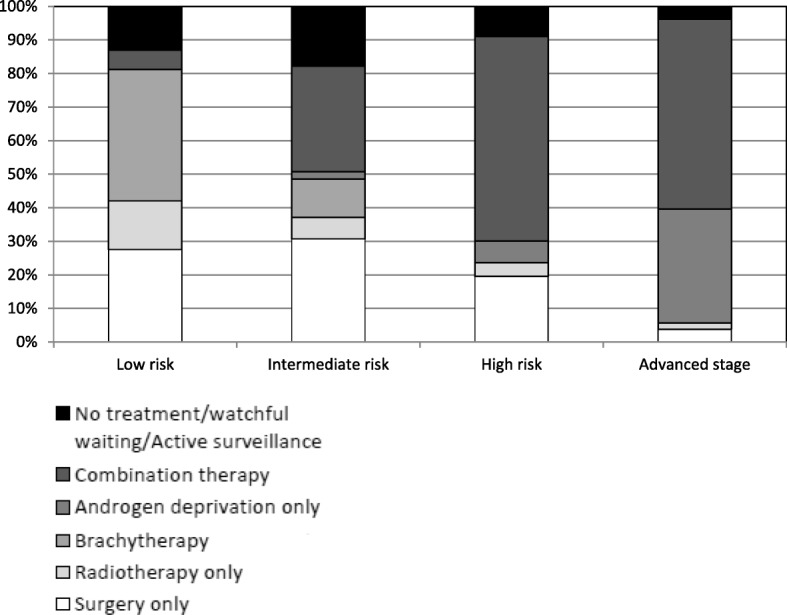
Analysis of treatments by prostate risk classification group

The different treatment combinations according to age groups and risk of disease progression are detailed in Table 3 and Fig. 2. Overall, 373 patients (82.5%) received at least one treatment, while 79 (17.5%) had non-invasive treatment, active surveillance or watchful waiting. Among patients treated with more than one therapeutic strategy, the most frequent combination was external radiotherapy with androgen deprivation (*n* = 102, 22.6%). There was a significant difference in treatment types according to age and prostate cancer risk group (p < 0.0001), with prostatectomy more frequently performed in patients aged < 75 years with localized cancer and at low or intermediate risk. Brachytherapy was more frequently used in patients aged < 65 years with localized cancer and at low risk. Androgen deprivation therapy was more frequent in high risk patients aged 75 and over, with advanced disease.

**Table 3:** Treatment according to age and risk classification (*N* = 452), Martinique, 2013

	All		≤ 64 years		65–74 years		≥ 75 years		*P*	Risk Classification									*P*
										Low risk		Intermediate risk		High risk		Regional/metastatic		Unknown	
	n	%	n	%	n	%	n	%		n	%	n	%	n	%	n	%	n	
	452	100.0	183	40.5	178	39.4	91	20.1		69	17.9	140	36.4	123	31.9	53	13.8	67	
Treatment									< 0.0001										< 0.0001
Surgery only	104	23.0	46	25.1	51	28.6	7	7.7		19	27.5	43	30.7	24	19.5	2	3.8	16	
Radical prostatectomy	93	89.4	42	91.3	47	92.2	4	57.1		17	89.5	42	97.7	23	95.8	2	100	9	
Transurethral resection	11	10.6	4	8.7	4	7.8	3	42.9		2	10.5	1	2.3	1	4.2	0	0	7	
External radiotherapy	26	5.7	6	3.3	11	6.2	9	9.9		10	14.5	9	6.4	5	4.1	1	1.9	1	
Brachytherapy	45	10.0	34	18.6	8	4.5	3	3.3		27	39.1	16	11.4	0	–	0	–	2	
Androgen deprivation only	34	7.5	7	3.8	9	5.1	18	19.8		0	–	3	2.1	8	6.5	18	33.9	5	
Combination therapy^a^	164	36.3	52	28.4	72	40.4	40	43.9		4	5.9	44	31.5	75	61.0	30	56.6	11	
No treatment / watchful waiting/ Active surveillance	79	17.5	38	20.8	27	15.2	14	15.4		9	13.0	25	17.9	11	8.9	2	3.8	32	

^a^Combination refers to any combination of surgery, radiation, chemotherapy, androgen deprivation, and/or chemotherapy

**Fig. 3:**
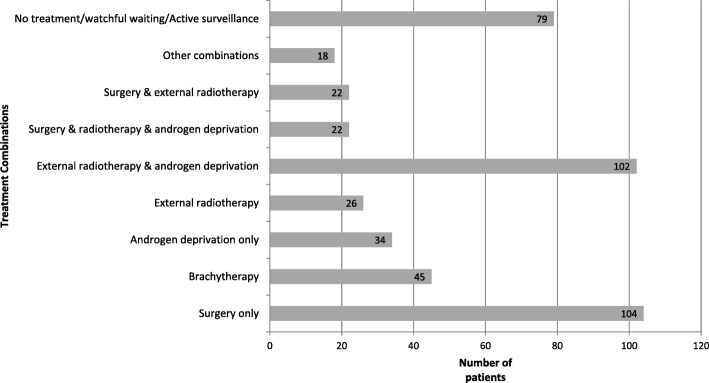
Details of the different treatment combinations used in the study population

No patient at low risk received androgen deprivation therapy. The different treatment combinations used in the study population are presented in Fig. 3.

For the year 2013, according to the data from our PBCR, it is estimated that less than 5% left Martinique to undergo treatment. The vast majority of patients with a de novo diagnosis were managed and treated by the cancer care teams on site in Martinique.

Only 9 patients (2%) travelled to mainland France for therapy; the remaining 98% were managed on site. Overall, 75% of all patients had a prostate MRI, 80.1% had a CT bone scan, and 60.8% had an abdominal CT scan. Prostate MRI was most frequently performed among patients aged < 65 years (*p* = 0.036) and at low risk (*p* = 0.017). CT bone scan was most frequently performed in patients at intermediate and high risk (*p* < 0.0001).

## Discussion

This is the first population-based study performed in the Caribbean on PCa management and treatment patterns in the French West-indies. Most PCa research studies in the Caribbean have evaluated cohorts from single referral institutions or were performed in highly selected patient groups, with limitations regarding the generalization of results, despite statistical quality control. Studies from population-based cancer registries represent a meaningful standard of comparison for the Caribbean zone, and may help to limit selection biases that could affect the results obtained.

In our study, three quarters of the patients had localized disease, and overall, a total of 79.2% were at intermediate (42.2%) or high risk (37.0%) suggesting that more than three quarters of patients diagnosed with localized disease may nonetheless have poor prognosis.

Regarding the population of older subjects, a total of 59.5% of patients in our study were aged 65 years and over. The main characteristics of these patients were a significantly higher proportion of patients with clinical symptoms (urinary or genital) at diagnosis, a higher risk of recurrence, and an increasing Gleason score with increasing age. Radical prostatectomy was less frequent in this group, which is understandable considering the life expectancy of the patients and the invasive nature of this treatment strategy.

Elderly patients aged ≥75 years were observed to be more often diagnosed with higher Gleason score and a higher risk of disease progression (37.8%). We also observed a significant difference in the treatment strategies according to age, stage and risk class. For example, radical prostatectomy was significantly more frequent in patients aged < 75 years, in those with localized cancer, and in those at low or intermediate risk. On the other hand, older patients (75+) were more likely to receive transurethral resection only (43% vs 9% among < 75 years old). The choice of therapy should not be based on chronological age only, but also on biological age and overall health status [18]. The stage distribution of PCa patients observed in our study is in line with those observed in mainland France, in Europe, and elsewhere in the Caribbean. One study, covering 11 French counties in 2001, showed that the proportion of localized PCa (T1 or T2) was 86.6%. The rate of use of invasive curative treatment (radical prostatectomy and radiotherapy) was 58.4% for localized cancers [34]. African-Caribbean studies on urological management of PCa in Trinidad and Tobago showed that most cases were found to be high risk (63.1%) followed by intermediate risk (29.6%) and low risk (7.3%) [35]. In this latter study, intermediate and high risk groups represented 92% of all cases diagnosed. This suggests that cases in Martinique are diagnosed at the same rate as these other countries, probably partly reflecting similar levels of population awareness, and health system infrastructure.

In our study, we observed that the treatments were in line with the recommendations of the French Urology Association, which stipulates the need for appropriate management in light of the risk of recurrence, as well as taking account of comorbidities, stage at diagnosis, age and patient preferences [21]. Patients at low risk of disease progression can benefit from watchful waiting or curative treatment for localized cancers, whereas in patients at intermediate risk, prostatectomy and radiotherapy are recommended as standard.

Other studies, based on clinical series in Martinique [36, 37], have shown a similar distribution of treatment approaches. Other studies have been performed in Guadeloupe in the framework of cohort studies or patient series [38–43]. Radiotherapy with androgen deprivation in high-risk PCa was studied among 59 consecutive patients and was found to be effective, as observed in other populations [41].

We found a significant difference in the treatment strategies according to age, stage and risk of disease progression. Indeed PCa management should be chosen in light of the risk of recurrence, comorbidity, stage, age and patient’s preference. Yet, reports to date suggest that active surveillance should be considered as the appropriate treatment for PCa surveillance for men at low-risk [44, 45]. A study in France showed that watchful waiting was proposed to 17.5% of the patients who were mostly young patients with localized disease and low risk. This choice is also an option in patients at low risk for whom invasive treatments can be reduced to a minimum. [34, 46, 47]. However, careful selection of patients who may be amenable to this strategy is indispensable in order to distinguish those who require active surveillance from those in whom it is possible to forego treatment [48–50].

In our study, prostate magnetic resonance imaging (MRI) was performed in 75% of patients, while computerized tomography (CT) bone scan was frequently performed in patients at intermediate to high risk, since it is the reference exam for the detection of bone metastasis. The diagnostic performance of MRI depends on the extent of disease and the tumour volume, with good sensitivity for Gleason scores ≥7. In case of a visible lesion, the tumour mapping achieved with MRI also makes it possible to adapt the treatment strategy. MRI is indeed the recommended imaging technique for assessing the extent of disease in PCa, suggesting that in our population this diagnostic recommendation was generally followed.

Unfortunately, Martinique does not yet dispose of a Positron Emission Tomography – Computed Tomography (PET-CT) device. An ongoing project planning to build a PET center in Martinique will be very useful in the future for PCa management in the whole Caribbean area.

Nuclear medicine imaging procedures and particularly PET-CT are useful in PCa management strategies. PET-CT using several radio-tracers such as 18F-Choline or recently 68Ga PSMA demonstrated a higher diagnostic efficacy compared with conventional imaging. In particular, 68Ga-PSMA PET/CT seems to be a promising tool for staging of primary prostate cancer and restaging after recurrence [51]. In biochemical relapse, 68Ga-PSMA PET imaging can increase detection of metastatic sites, even at low serum PSA levels.

The strength of this study is the fact that this is a population-based study including all patients diagnosed in Martinique in 2013. Only 21 cases were excluded, in order to avoid bias in the analysis linked to the presence of multiple tumours.

Among the limitations of our study, it should be noted that we had difficulty accessing data about the stage of disease at the time of diagnosis. Indeed, the cancer registrars may have difficulty extracting the full TNM code from clinical records, if this has not been explicitly recorded by the clinicians or pathologists. Furthermore, regarding access to data concerning the treatment administered or dispensed, treatment administered in-hospital can be identified from data sources already used in the registry (e.g. medical informatics databases from the national social security system). However, in our study, we did not have data regarding treatment performed more than 2 years after diagnosis, and this represents a potential limitation.

For patients who travel outside of Martinique for treatment, data on treatment were reported in the medical records. In our study, only 9 patients (2%) travelled to mainland France for therapy; the remaining 98% were managed on site. Due to the fact that the data was derived from the integrated national informatics system for the French social security system, we were able to confirm the rate of patients who travel outside Martinique for treatment.

This study adds valuable information on prostate cancer patterns of diagnosis in the Caribbean region. This is especially important considering the high incidence and mortality of the disease in the region. Unlike our study, most PCa research studies in the Caribbean have evaluated cohorts from single referral institutions or were performed in highly selected patient groups, with limitations regarding the generalization of results, despite statistical quality control. In view of the paucity of data from the Caribbean region on this topic, comparisons with other countries from the Caribbean area are limited by the accuracy and quality of data from the countries that are not covered by population-based cancer registries. Our study is descriptive by nature and does not serve to draw causal relations; nonetheless, it can serve as a benchmark for future analyses of overall and net survival according to the diagnostic and therapeutic strategies.

In order to better coordinate public health policy, the Regional Health Agencies (Agence Regionale de Santé, ARS) need epidemiological data, with a view to implementing appropriate anti-cancer programmes, with suitable oversight and indicators for evaluating success. Our study will help to meet the needs of the ARS in the French overseas territories, in particular by proposing and describing indicators that are adapted to local and regional needs in terms of healthcare, for use in future public health policies for this region. This work will contribute to identifying public health challenges in our geographical region, especially to assess the adequacy of the healthcare opportunities offered to our populations.

## Conclusions

This study from the Martinique Cancer Registry provides the most comprehensive clinical data regarding the quality of diagnosis and management of patients with PCa in Martinique. The diagnostic and therapeutic management was found to be in line with international recommendations, except for PET CT imaging. These findings will contribute to identifying public health challenges in the management of PCa in this geographic area. Genomic studies on PCa patients could help to better understand clinical factors associated with high incidence of prostate cancer in Martinique and help develop better treatment strategies. The future installation of a PET-CT scan in Martinique will enable better staging of prostate cancers and optimized therapeutic follow-up of this disease. Finally, the results of our study will contribute to improving epidemiological knowledge of cancer patterns in ultra-peripheral geographic regions, and help to develop surveillance tools and raise awareness of health states in these populations.

## Abbreviations

CI: Confidence interval
CT-scan: Computed tomography scan
MCR: Martinique Cancer Registry
MRI: Magnetic resonance imaging
PBCR: Population-based cancer registries
PCa: Prostate cancer
PET-CT: Positron Emission Tomography – Computed Tomography
PSA: Prostate-specific antigen
